# Sex and Age Differences in the Association Between Social Determinants of Health and Cardiovascular Health According to Household Income Among Mongolian Adults: Cross-Sectional Study

**DOI:** 10.2196/44569

**Published:** 2023-12-01

**Authors:** Sun Young Shim, Hyeonkyeong Lee

**Affiliations:** 1 College of Nursing Yonsei University Seoul Republic of Korea; 2 Mo-Im Kim Nursing Research Institute College of Nursing Yonsei University Seoul Republic of Korea

**Keywords:** social determinants of health, cardiovascular health, education, household income, health insurance, association, risk factors, cardiovascular, cardiovascular disease, cross-sectional study

## Abstract

**Background:**

Although social determinants of health (SDH) are an underlying cause of poor cardiovascular health (CVH), there is insufficient evidence for the association between SDH and CVH, which varies by sex and age among Mongolian adults.

**Objective:**

We aimed to explore whether education, household income, and health insurance were associated with CVH according to sex and age among Mongolian adults.

**Methods:**

The final sample included data on 5691 participants (male: n=2521. 44.3% and female: n=3170, 55.7%) aged 18-69 years from the 2019 World Health Organization STEPwise approach to noncommunicable disease risk-factor surveillance. CVH was measured using a modified version of Life’s Simple 7 with 4 health behaviors (cigarette smoking, BMI, physical activity, and a healthy diet) and 3 biological factors (blood pressure, fasting glucose, and total cholesterol blood levels) and classified into poor, intermediate, and ideal levels as recommended by the American Heart Association. Multinomial logistic regression analyses examined the associations between SDH and CVH by monthly equivalized household income after adjusting for age, sex, work status, area, history of myocardial infarction or stroke, use of aspirin, and use of statin. Subgroup analyses were conducted to examine the associations between SDH and CVH based on sex and age, considering monthly equivalized household income as a key variable.

**Results:**

Using the ideal level of CVH as a reference, among those with the lowest household income, having less than 12 years of education, and not having health insurance were associated with poor CVH (education level: odds ratio [OR] 2.42, 95% CI 1.30-4.51; *P*=.006; health insurance: OR 2.17, 95% CI 1.13-4.18; *P*=.02). These associations were more profound among female individuals (education level: OR 2.99, 95% CI 1.35-6.63; *P*=.007; health insurance: OR 2.54, 95% CI 1.09-5.90; *P*=.03) and those aged 18-44 years (education level: OR 3.22, 95% CI 1.54-6.72; *P*=.002; health insurance: OR 2.03, 95% CI 0.98-4.18; *P*=.06).

**Conclusions:**

Participants in the lowest household income group with lower educational levels and without health insurance were more likely to have poor CVH, and these results were more pronounced in female individuals and young adults. These findings suggest the need to develop strategies for CVH equity in Mongolian female individuals and young adults that consider income levels, education levels, and health insurance.

## Introduction

The trend toward universal health coverage in Mongolia seems to have stagnated. Service coverage related to cardiovascular health (CVH) risk factors such as hypertension, diabetes, and smoking is also below the global average; there was no improvement between 2000 and 2019 [1]. Of the 4 noncommunicable diseases (NCDs) most common among Mongolian adults aged 30-70 years, cardiovascular disease (CVD) accounted for the largest proportion (17.3%) of deaths [2]. In a cross-sectional study that examined a nationally representative sample of 70,380 people aged 35-75 years in Inner Mongolia, 25% of the study participants were at high-risk for CVD, with more than 10% at risk of developing CVD after 10 years [3]. However, Mongolian adults’ low rates of hypertension control treatment, along with high rates of smoking, sodium intake, and obesity, indicate that CVH is not adequately managed in Mongolian adults [4-7].

There is substantial evidence for the effectiveness of individual lifestyle changes to reduce CVH risk [8-10]; nevertheless, it is necessary to examine situations such as the structural social determinants of health (SDH) in Mongolia, where essential service coverage is not guaranteed. SDH, such as education and health insurance coverage, can be more important than lifestyle choices in influencing population health and health equity because of their connection to CVD pathogenesis. Chronic psychosocial stress can be induced among individuals who are susceptible to SDH, and this stress can lead to chronic inflammation by activating the sympathetic nervous system linked to altered stress hormone. For example, glucocorticoid receptor resistance due to chronic activation of the sympathetic nervous system blunts the anti-inflammatory response; it could contribute to enabling CVD development and progression. [11]. Further, the Denmark study showed that a low education level was independently associated with a higher risk of CVD among 1.6 million Danish employees [12]. In Colombia, people with lower education levels have a higher risk of dying prematurely from CVD, and these education inequalities affect female individuals more than male individuals [13]. A prospective cohort study that examined 390,881 Chinese adults aged 18-64 years with a mean follow-up of 10.4 years revealed that having private health insurance coverage was associated with a 21% lower risk of CVD mortality compared to being uninsured [14].

However, there are insufficient studies showing the influence of SDH on CVH among Mongolian adults. Thus, the identification of SDH should be the foundation for developing strategies that promote CVH and reduce long-term health inequality [15]. Research on this topic can provide critical information that can be used for evidence-based planning and decision-making based on priorities regarding CVH equity. As prevalence of CVD risk differed according to sex and age among Mongolian adults [3,4], the population group characteristics should be considered when confirming the influence of SDH on CVH. Thus, this study aimed to explore the sex and age differences in the independent associations between SDH (educational level and health insurance) and CVH among Mongolian adults.

## Methods

### Data and Study Participants

Data were obtained from the 2019 Mongolia STEPwise approach to noncommunicable disease risk-factor surveillance (STEPS). The World Health Organization (WHO) initiated STEPS in various countries, recognizing a need for standardized worldwide risk-factor data on NCD risk factors for surveillance systems [16,17]. STEPS was conducted in Mongolia to assess the impact and effect of the integrated national program on “NCD Prevention and Control” [5]. STEPS is a repeated household survey that is population based and cross-sectional. It uses multistage cluster sampling to extract and investigate a nationally representative sample [18]. The fourth Mongolia STEPS survey was carried out using 377 sampling clusters selected from 21 provinces and 9 districts of Ulaanbaatar in 2019 [5]. Of the 6654 people who participated in the 2019 Mongolia STEPS, participants aged younger than 18 years (n=173, 2.6%) and those who had missing data on key variables (n=790, 11.9%) were excluded and 5691 (85.5%) participants were finally included in this secondary data analysis.

### Ethical Considerations

All participants provided written informed consent prior to participating in the 2019 Mongolia STEPS. This study was granted an exemption by the institutional review board (4-2021-1341) of Severance Hospital at Yonsei University College of Medicine, Republic of Korea, which allows secondary analyses without additional consent.

### SDH Factor

Information about education level, possessing health insurance, and average household earnings in the past year were assessed through face-to-face interviews [16]. Educational attainment was categorized into 2 groups: <12 years of education and ≥12 years of education (reference group). Current health insurance was assessed using the question “Do you currently have any kind of health insurance or health care coverage?” [16]. The average monthly household income was calculated by dividing the average household income in the previous year by 12. Equivalized household income was defined as the average monthly household income divided by the square root of the number of household members [19]. The study participants were divided into 4 groups according to the quartiles of equivalized household income.

### CVH Factor

We used the modified Life’s Simple 7 by the American Heart Association to measure CVH. It addresses 4 health behaviors, including BMI, smoking, physical activity, and diet, and 3 health risk factors, including blood pressure, fasting glucose levels, and total cholesterol [20]. A cutoff of 23 kg/m^2^ for BMI, which has been validated for the Asian population, was used in this study [21]. Smoking, physical activity, and diet were assessed through face-to-face interviews using standardized questionnaires [16]. Salt consumption for diet was used instead of the American Heart Association’s guidelines [22]. Blood pressure was measured 3 times for 3-minute intervals. Each measurement was taken on the left arm, with the participants in a seated posture [18]. The mean of the last 2 measurements was used in the analysis [18]. Overnight fasting blood samples were collected at a local clinic or health center [18]. CVH metrics were scored as poor (0 points), intermediate (1 point), and ideal (2 points). The total CVH scores were calculated as the sum of the scores for each component for a possible range of 0-14 (Table S1 in Multimedia Appendix 1). Scores of 0-7 were considered as poor, 8-11 as intermediate, and 12-14 as ideal for CVH [23].

### Covariates

We adjusted for sex, age (18-39, 40-59, and ≥60 years), work status (employee, self-employed, or others), area (rural or urban), history of myocardial infarction or stroke (yes or no), use of aspirin (yes or no), and use of statin (yes or no). The history of myocardial infarction or stroke was investigated using the question “Have you ever had a heart attack or chest pain from heart disease (angina) or a stroke (cerebrovascular accident or incident)?” The use of aspirin or statin was assessed using the questions “Are you currently taking aspirin regularly to prevent or treat heart disease?” and “Are you currently taking statins (Lovastatin/Simvastatin/Atorvastatin or any other statin) regularly to prevent or treat heart disease?” respectively. The covariate data were collected by self-reporting.

### Statistical Analysis

Descriptive values were calculated for the total participant group as well as the male and female groups, represented as a weighted mean (95% CI) and number (weighted proportion). The differences in variables according to CVH categories in male and female participants were calculated using ANOVA for continuous variables and the chi-square test for categorical variables. Independent associations between SDH (education level and health insurance) and CVH were assessed through multinomial logistic regression analyses between the sexes after adjusting for covariates. Subgroup analyses regarding associations between SDH and CVH according to monthly equivalized household income were conducted among the total participants. This subgroup analysis according to monthly equivalized household income was also performed by sex and age to identify different sex and age associations between SDH and CVH by household income. The period of public education in Mongolia gradually changed from 10 years in 2004 to 12 years in 2013; students who graduated in 2013 were the first to have received 12 years of public education. Therefore, sensitivity analyses were conducted by defining low education level as less than 11 years of education and examining the association between education level and CVH according to monthly equivalized household income. Adjusted odds ratios (ORs) and 95% CIs were calculated using the PROC SURVEYLOGISTIC procedures to apply population weights, and all statistical tests were performed using SAS (version 9.4; SAS Institute Inc).

## Results

The general characteristics of study participants by sex according to CVH are described in Multimedia Appendix 2. Of the total 5691 participants, 29.6% (n=1685), 56.4% (n=3208), and 14% (n=798) showed poor, intermediate, and ideal CVH, respectively. The mean CVH score of study participants was 8.80 (SD 2.46). The weighted mean age of study participants was 37.5 (SE 0.26) years, and more than two-thirds (2888/5691, 69.3%) were aged 18-44 years. Approximately half (n=3075, 49.6%) of the participants had less than 12 years of education, and one-fifth (n=888, 19.5%) had no health insurance. In male participants, 41.1% (n=1036), 50.2% (n=1265), and 8.7% (n=220) showed poor, intermediate, and ideal CVH, respectively. The mean age, the proportion of employees, self-employment, and use of aspirin were likely to be higher in participants with poor CVH (all *P*<.001). In female participants, 20.5% (n=649), 61.3% (n=1943), and 18.2% (n=578) showed poor, intermediate, and ideal CVH, respectively. The mean age and the proportion of aspirin and of statin use were likely to be higher in those with poor CVH (all *P*<.001). Those with less than 12 years of education were more likely to have poorer CVH (*P*=.001).

Table 1 shows the results of multinomial logistic regression regarding the association between education, health insurance, and CVH categories. Of the total participants, using ideal CVH as a reference, those with less than 12 years of education were not associated with intermediate and poor CVH after adjusting for potential covariates (intermediate CVH: OR 1.12, 95% CI 0.91-1.40; poor CVH: OR 1.00, 95% CI 0.78-1.29). Similarly, of the total participants, those without health insurance were not associated with intermediate and poor CVH, respectively (intermediate CVH: OR 0.97, 95% CI 0.73-1.28; poor CVH: OR 1.19, 95% CI 0.85-1.67). For both male and female participants, low education level and absence of health insurance were not associated with intermediate and poor CVH.

**Figure 1:**
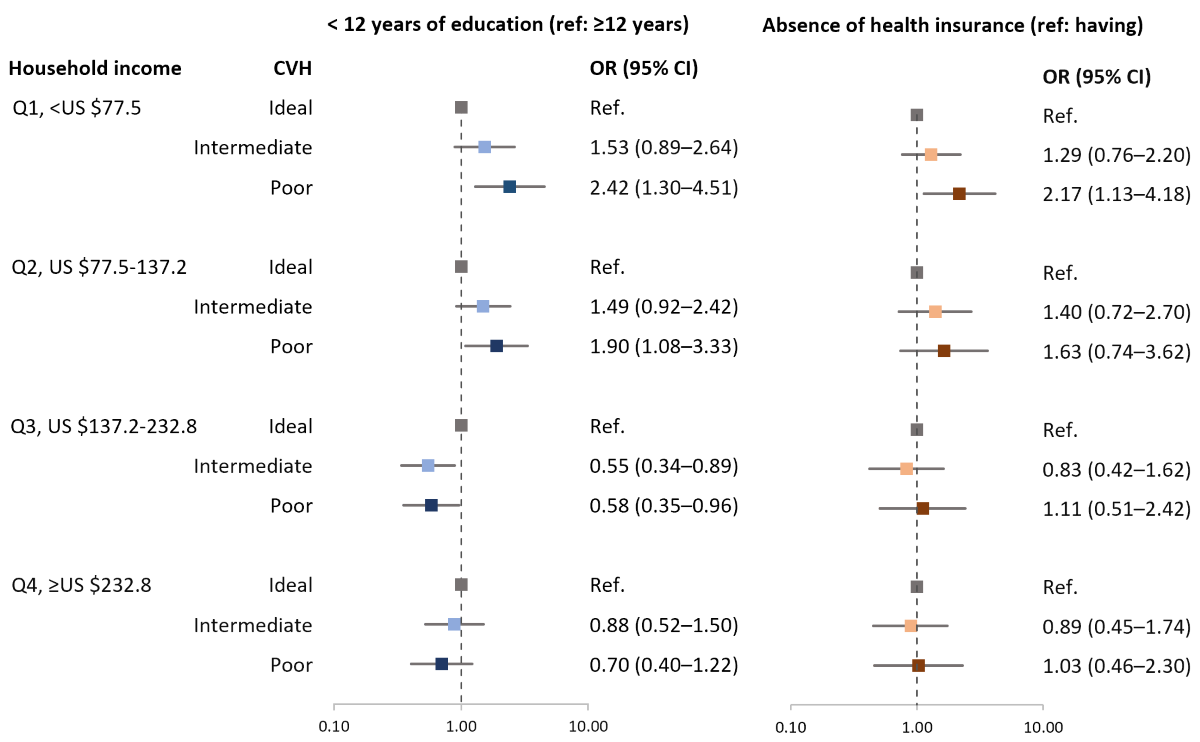
Results of multinomial logistic regression of the association between social determinants of health and CVH according to household income. Age, sex, work status, area, history of myocardial infarction or stroke, use of aspirin, and use of statin were adjusted. CVH: cardiovascular health; OR: odds ratio; Ref: reference.

However, as a result of stratification according to quartiles of monthly household income, participants with less than 12 years of education in the Q1 (lowest household income) and Q2 groups were associated with a 2.42 (95% CI 1.30-4.51) and 1.90 (95% CI 1.08-3.33) times higher likelihood of poor CVH, respectively (Figure 1 and Table S2 in Multimedia Appendix 1). These associations were consistently found when defining low education level as less than 11 years (data not shown). Participants without health insurance in the Q1 group were also associated with a 2.17 (95% CI 1.13-4.18) times higher likelihood of poor CVH.

**Table 1:** Results of multinomial logistic regression of the association between education, health insurance, and CVH^a^ by modified Life's Simple 7^b,c^.

Variables			Intermediate CVH			Poor CVH	
			n (%)	OR^d^ (95% CI)	n (%)		OR (95% CI)
**Education**							
	**All participants (N=5691)**						
		≥12 years	1458 (55.7)	1.00 (N/A^e^)	721 (27.6)		1.00 (N/A)
		<12 years	1750 (56.9)	1.12 (0.91-1.40)	964 (31.4)		1.00 (0.78-1.29)
	**Male participants (n=2521)**						
		≥12 years	497 (50.0)	1.00 (N/A)	405 (40.7)		1.00 (N/A)
		<12 years	768 (50.3)	0.98 (0.6-1.40)	631 (41.4)		0.95 (0.64-1.40)
	**Female participants (n=3170)**						
		≥12 years	961 (59.3)	1.00 (N/A)	316 (19.5)		1.00 (N/A)
		<12 years	982 (63.4)	1.24 (0.94-1.64)	333 (21.5)		0.97 (0.68-1.38)
**Health insurance**							
	**All participants (N=5691)**						
		Yes	2718 (56.6)	1.00 (N/A)	1415 (29.5)		1.00 (N/A)
		No	490 (55.2)	0.97 (0.73-1.28)	270 (30.4)		1.19 (0.85-1.67)
	**Male participants (n=2521)**						
		Yes	1014 (50.1)	1.00 (N/A)	847 (41.8)		1.00 (N/A)
		No	251 (50.7)	0.86 (0.56-1.33)	189 (38.2)		1.00 (0.63-1.58)
	**Female participants (n=3170)**						
		Yes	1704 (61.4)	1.00 (N/A)	568 (20.5)		1.00 (N/A)
		No	239 (60.8)	1.05 (0.75-1.49)	81 (20.6)		1.47 (0.95-2.27)

^a^CVH: cardiovascular health.

^b^The estimates are in reference to the ideal CVH group.

^c^Age, sex, work status, area, history of myocardial infarction or stroke, use of aspirin, and use of statin were adjusted.

^d^OR: odds ratio.

^e^N/A: not available.

**Figure 2:**
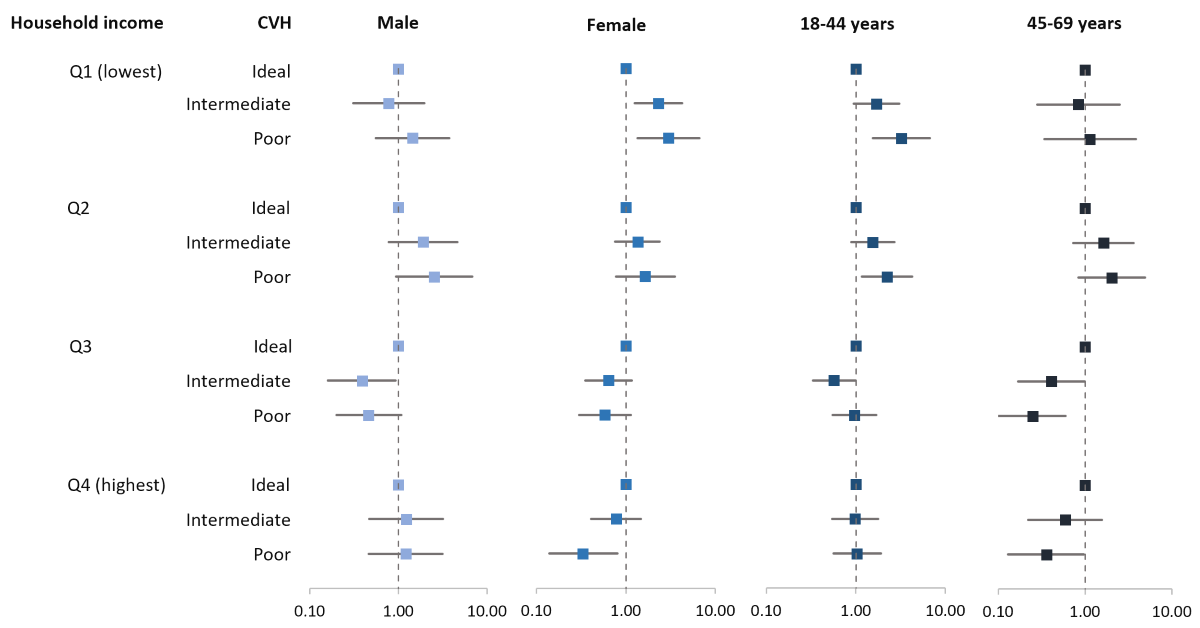
Results of multinomial logistic regression of the association between low education level and CVH according to household income by sex and age. The reference is ≥12 years of education. Adjusted for all other factors not involving the subgroup, including age, sex, work status, area, history of myocardial infarction or stroke, use of aspirin, and use of statin were adjusted. CVH: cardiovascular health.

The associations between education level and CVH according to monthly household income quartile by sex and age are illustrated in Figure 2. Female participants in the Q1 (lowest household income) group with less than 12 years of education were associated with a 2.99 (95% CI 1.35-6.63) times higher likelihood of poor CVH compared to female participants with more than 12 years of education (Table S3 in Multimedia Appendix 1). However, male participants with less than 12 years of education were not associated with intermediate and poor CVH in the Q1 group (intermediate CVH, OR 0.78, 95% CI 0.31-1.96; poor CVH, OR 1.44, 95% CI 0.56-3.72). Participants in the Q1 group who were aged 18-44 years and had less than 12 years of education were also associated with a 3.22 (95% CI 1.54-6.72) times higher likelihood of poor CVH. Participants in the Q3 and Q4 groups, aged 45-69 years, with less than 12 years of education were associated with a lower likelihood of poor CVH (Q3, OR 0.25, 95% CI 0.10-0.60; Q4, OR 0.36, 95% CI 0.13-0.97).

**Figure 3:**
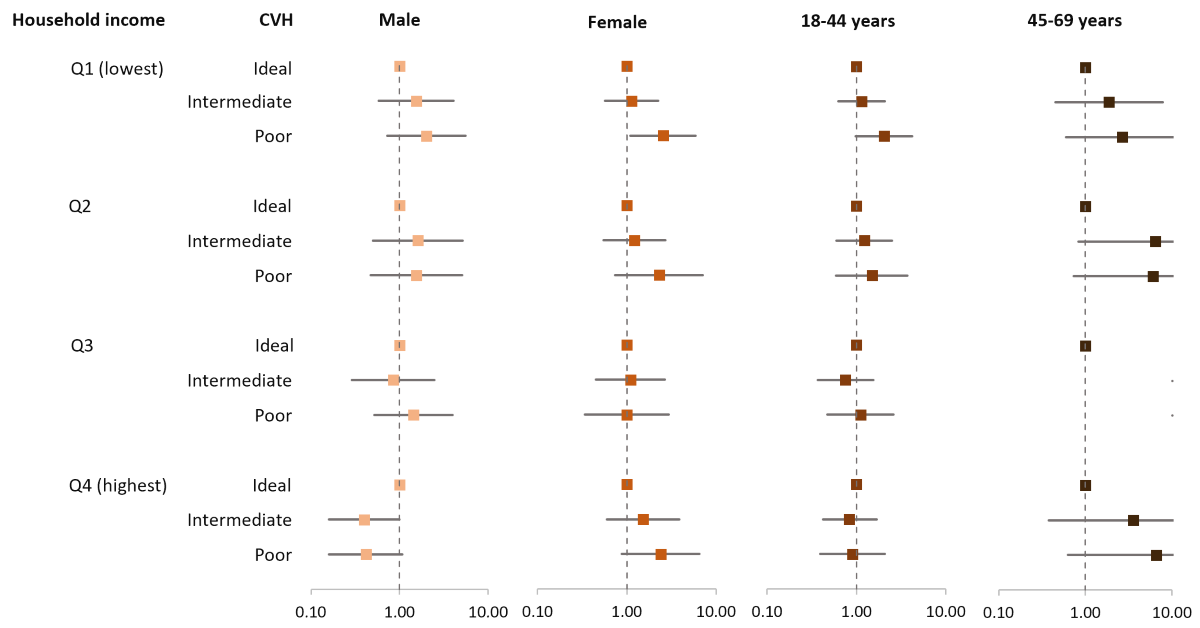
Results of multinomial logistic regression of the association between absence of health insurance and CVH according to household income by sex and age. The reference is having health insurance. Adjusted for all other factors not involving the subgroup, including age, sex, work status, area, history of myocardial infarction or stroke, use of aspirin, and use of statin were adjusted. CVH: cardiovascular health.

Similarly, female participants in the Q1 (lowest household income) group without health insurance were associated with a 2.54 (95% CI 1.09-5.90) times higher likelihood of poor CVH compared to female participants with health insurance (Figure 3 and Table S4 in Multimedia Appendix 1). However, male participants in the Q1 group without health insurance were not associated with poor CVH (OR 2.02, 95% CI 0.73-5.57). Participants in the Q1 group, aged 18-44 years, without health insurance were marginally associated with a 2.03 (95% CI 0.98-4.18) times higher likelihood of poor CVH. Participants in the Q1 group, aged 45-69 years, without health insurance were not associated with poor CVH (OR 2.67, 95% CI 0.60-11.95). The OR for the Q3 group of participants aged 45-69 years was not accounted for as the model did not converge due to quasi-complete separation.

## Discussion

### Principal Findings

For all participants, having less education or no health insurance was not associated with CVH; however, stratified analyses by monthly household income quartiles showed that having less education or no health insurance was associated with poor CVH in the lowest household income groups. These CVH disparities were more pronounced among female individuals and young adults. This study contributes to establishing policies implementation that reduces the disparity of CVH, the most notable cause of disease burden, by presenting the basis for prioritizing a vulnerable population. For example, health service delivery guidelines should include consideration of the link between SDH and CVD, and training for health care providers should emphasize this. Low levels of education may be associated with low levels of health literacy for CVD prevention and management practices [24,25]. Therefore, health care providers should assess the health literacy levels of their target population and provide interventions that take this into account.

There was a gap in the prevalence of CVD risk factors according to the WHO region or income level; Mongolia is a country with a high prevalence of CVD risk factors **[26]**. For example, nonelevated blood pressure, one of the tracer indicators for calculating the universal health coverage service coverage index, was 29, indicating the low coverage of blood pressure management in Mongolia **[26]**. Further, the linear trends in cardiovascular risk factors such as smoking, obesity, and elevated blood pressure among Mongolian adults are all projected to be significantly higher than the global target by 2025 **[27]**. Thus, there is a need for the adoption and dissemination of cardiovascular risk-factor management guidelines. The WHO-initiated STEPS in 2002 is practical and helpful in monitoring the status of CVH and can identify the achievement rate for each cardiovascular risk factor, thereby aiding the formulation of effective health policies for managing risk factors at a country level **[16]**. Establishing a high-vulnerability priority population in population health is essential for ensuring universal health in which no one is excluded.

The association between education level and CVH is inconsistent in previous studies. A cross-sectional study of 7771 nationally representative participants aged ≥25 years in the United States reported that participants with at least a college degree were 4.12 times more likely to have ideal CVH status than participants with less than a high school education **[28]**. However, a recent cross-sectional study of 1634 Asian American adults aged ≥20 years in the United States revealed that low educational attainment was associated with a low likelihood of having ideal CVH compared to high educational attainment after adjusting for age and sex; however, this association became nonsignificant after additionally adjusting for income category and nativity status **[26]**. Further studies are needed to comprehensively understand the relationship between educational attainment and CVH in the contexts of including other SDH, such as income and race, for health equity in populations that are vulnerable to CVH problems.

Mongolia implemented a social health insurance system in 1994 to maintain equity in access to health care. Consequently, 95.6% of the population has been covered by social health insurance since 2017 **[29,30]**. Despite this high health insurance coverage rate, health expenses are significantly related to poverty in Mongolia, with pronounced consequences in households with patients with NCD **[31]**. Our study also showed that the population without health insurance had a higher risk of poor CVH status in the lowest income group than in the higher income groups, which is consistent with the previous study **[32]**.

In this study, the CVH disparities according to SDH were predominant in female individuals and young adults. Mongolia is ranked 69th out of 156 countries in the global gender gap index, and the score of estimated earned income among the subitems measuring this index was 0.67 (0.00=imparity, 1.00=parity) **[33]**. Numerous previous studies support the sex differences in cardiovascular risk factors and diseases **[34-36]**. Our study revealed that low education level or lack of health insurance contributed more to poor CVH, especially among low-income female individuals. Furthermore, a history of CVD in Mongolians currently appears in relatively young population groups; an increase in CVD at a young age is a remarkable health issue **[5]**. Therefore, efforts to promote CVH that consider sex and age, such as interventions for modifying CVH for young adults or creating strategies that target gender equality, might be needed to achieve health equity in Mongolia. In countries with limited universal health coverage such as Mongolia, a strategy of using text messages to target younger populations may be useful in managing hypertension to prevent CVD, as identified in a previous research review [37].

The strength of this study was that, to the best of our knowledge, this study is the first to identify the sex and age differences in the association between SDH and CVH according to income among Mongolian adults. Further, we used nationally representative Mongolian data to minimize external validity threats.

### Limitations

This study has some limitations. First, it is possible that participants who had less than 12 years of education but completed all public education courses were grouped together with those who did not complete public education courses. This can be attributed to the gradual change of public education in Mongolia from 10 years in 2004 to 12 years in 2013. Thus, we performed the sensitivity analysis by dividing the education level based on 11 years and confirmed that the results were similar to our primary results. Second, we measured salt consumption as the diet component of CVH, which is not a guideline in Life’s Simple 7. Third, there is a possibility of misclassification of self-reported variables. Fourth, we used the most recent fourth survey to determine the latest findings on the association between SDH and CVH. The STEPS Mongolia cross-sectional survey began in 2005 and has been conducted 4 times every 4 to 6 years. Further research is needed to identify trends in CVH following structural SDH in order to prioritize risk factors that have not been improved over time. Fifth, the STEPS data did not provide information on individual residence, limiting our ability to determine the impact of residence on health. Although we found that living in a rural area was associated with lower CVH compared with urban area, we suggest including variables related to residence because even within the same rural area, there may be differences in the environment in which people live (Table S5 in Multimedia Appendix 1). Lastly, although we adjusted potential cofounders, there might still be a chance of residual confounding we did not control for.

### Conclusions

In conclusion, we confirmed that in Mongolia, where universal health coverage is low, the population group that has low structural socioeconomic levels and health care coverage is more vulnerable to CVH problems. As in other low-income countries, female individuals are more vulnerable to poor CVH than male individuals, suggesting that this vulnerability can only be improved when fundamental problems such as structural health determinants are addressed. Strategies and interventions aimed at reducing the CVH disparity in the Mongolian population should be prioritized for female individuals and young adults (aged 18-44 years) with low socioeconomic status and without health insurance.

## Appendix

Multimedia Appendix 1 Supplementary table 1-5.

Multimedia Appendix 2 General characteristics of study participants according to cardiovascular health by the modified Life's Simple 7.

## Abbreviations

CVH: cardiovascular health
CVD: cardiovascular disease
NCD: noncommunicable disease
OR: odds ratio
SDH: social determinants of health
STEPS: STEPwise approach to noncommunicable disease risk-factor surveillance
WHO: World Health Organization

## Data Availability

The data sets generated or analyzed during this study are available in The World Health Organization noncommunicable disease microdata repository (World Health Organization, June 20, 2023, STEPwise approach to noncommunicable disease risk-factor surveillance 2019 Mongolia).

## References

[1] (2022). Global health observatory data repository. World Health Organization.

[2] Health indicators-2019. Center for Health Development.

[3] Xi Y, Cao N, Niu L, Zhu H, Bao H, Qiao L, Ji S, Yan T, Xu X, Wang W, Zhang X (2021). Prevalence and treatment of high cardiovascular disease risk in Inner Mongolia, China. Rev Cardiovasc Med.

[4] Potts H, Baatarsuren U, Myanganbayar M, Purevdorj B, Lkhagvadorj BU, Ganbat N, Dorjpalam A, Boldbaatar D, Tuvdendarjaa K, Sampilnorov D, Boldbaatar K, Dashtseren M, Batsukh B, Tserengombo N, Unurjargal T, Palam E, Bosurgi R, So G, Campbell NRC, Bungert A, Dashdorj N, Dashdorj N (2020). Hypertension prevalence and control in Ulaanbaatar, Mongolia. J Clin Hypertens (Greenwich).

[5] World Health Organization (2019). Fourth national steps survey on the prevalence of noncommunicable disease and injury risk factors-2019-2020. WHO Representative Office for Mongolia.

[6] Tuvdendorj A, Feenstra T, Tseveen B, Buskens E (2020). Smoking-attributable burden of lung cancer in Mongolia a data synthesis study on differences between men and women. PLoS One.

[7] Chimeddamba O, Gearon E, Stevenson C, Ng WL, Baasai B, Peeters A (2016). Trends in adult overweight and obesity prevalence in Mongolia, 2005-2013. Obesity (Silver Spring).

[8] Chung MK, Eckhardt LL, Chen LY, Ahmed HM, Gopinathannair R, Joglar JA, Noseworthy PA, Pack QR, Sanders P, Trulock KM (2020). Lifestyle and risk factor modification for reduction of atrial fibrillation: a scientific statement from the American Heart Association. Circulation.

[9] Lee LL, Mulvaney CA, Wong YKY, Chan ES, Watson MC, Lin HH (2021). Walking for hypertension. Cochrane Database Syst Rev.

[10] Blumenthal JA, Hinderliter AL, Smith PJ, Mabe S, Watkins LL, Craighead L, Ingle K, Tyson C, Lin PH, Kraus WE, Liao L, Sherwood A (2021). Effects of lifestyle modification on patients with resistant hypertension: results of the TRIUMPH randomized clinical trial. Circulation.

[11] Powell-Wiley TM, Baumer Y, Baah FO, Baez AS, Farmer N, Mahlobo CT, Pita MA, Potharaju KA, Tamura K, Wallen GR (2022). Social determinants of cardiovascular disease. Circ Res.

[12] Framke E, Sørensen JK, Andersen PK, Svane-Petersen AC, Alexanderson K, Bonde JP, Farrants K, Flachs EM, Hanson LLM, Nyberg ST, Villadsen E, Kivimäki M, Rugulies R, Madsen IEH (2020). Contribution of income and job strain to the association between education and cardiovascular disease in 1.6 million Danish employees. Eur Heart J.

[13] Jimenez M, Arroyave I (2020). How educational inequalities in cardiovascular mortality evolve while healthcare insurance coverage grows: Colombia, 1998 to 2015. Value Health Reg Issues.

[14] Song L, Wang Y, Chen B, Yang T, Zhang W, Wang Y (2020). The association between health insurance and all-cause, cardiovascular disease, cancer and cause-specific mortality: a prospective cohort study. Int J Environ Res Public Health.

[15] Marmot M, Friel S, Bell R, Houweling TAJ, Taylor S, Commission on Social Determinants of Health (2008). Closing the gap in a generation: health equity through action on the social determinants of health. Lancet.

[16] Riley L, Guthold R, Cowan M, Savin S, Bhatti L, Armstrong T, Bonita R (2016). The World Health Organization STEPwise approach to noncommunicable disease risk-factor surveillance: methods, challenges, and opportunities. Am J Public Health.

[17] (2022). STEPwise approach to NCD risk factor surveillance (STEPS). World Health Organization.

[18] (2005). WHO STEPS surveillance manual: the WHO STEPwise approach to chronic disease risk factor surveillance. Report no.: 9241593830. World Health Organization.

[19] (2005). What are equivalence scales?. Organisation for Economic Copperation and Development (OECD).

[20] Lloyd-Jones DM, Hong Y, Labarthe D, Mozaffarian D, Appel LJ, Van Horn L, Greenlund K, Daniels S, Nichol G, Tomaselli GF, Arnett DK, Fonarow GC, Ho PM, Lauer MS, Masoudi FA, Robertson RM, Roger V, Schwamm LH, Sorlie P, Yancy CW, Rosamond WD (2010). Defining and setting national goals for cardiovascular health promotion and disease reduction: the American Heart Association's strategic impact goal through 2020 and beyond. Circulation.

[21] WHO Expert Consultation (2004). Appropriate body-mass index for Asian populations and its implications for policy and intervention strategies. Lancet.

[22] Liu H, Yao Y, Wang Y, Ma L, Liu X, Guo S, Feng X, Chen Y, Chen X, Liu Z, Ji L, Li D, Zhou Y (2019). Ideal cardiovascular health metrics and the risk of non-alcoholic fatty liver disease: a cross-sectional study in Northern China. Liver Int.

[23] Corlin L, Short MI, Vasan RS, Xanthakis V (2020). Association of the duration of ideal cardiovascular health through adulthood with cardiometabolic outcomes and mortality in the Framingham offspring study. JAMA Cardiol.

[24] Kanejima Y, Shimogai T, Kitamura M, Ishihara K, Izawa KP (2022). Impact of health literacy in patients with cardiovascular diseases: a systematic review and meta-analysis. Patient Educ Couns.

[25] Lindahl B, Norberg M, Johansson H, Lindvall K, Ng N, Nordin M, Nordin S, Näslund U, Persson A, Vanoli D, Schulz PJ (2020). Health literacy is independently and inversely associated with carotid artery plaques and cardiovascular risk. Eur J Prev Cardiol.

[26] Alam MT, Echeverria SE, DuPont-Reyes MJ, Vasquez E, Murillo R, Gonzalez T, Rodriguez F (2021). Educational attainment and prevalence of cardiovascular health (Life's Simple 7) in Asian Americans. Int J Environ Res Public Health.

[27] (2018). Noncommunicable diseases Mongolia. World Health Organization.

[28] Johnson AE, Herbert BM, Stokes N, Brooks MM, Needham BL, Magnani JW (2022). Educational attainment, race, and ethnicity as predictors for ideal cardiovascular health: from the National Health and Nutrition Examination Survey. J Am Heart Assoc.

[29] Mongolia health indicators 2017. World Health Organization Regional Office for the Western Pacific (WPRO-WHO).

[30] Dorjdagva J, Batbaatar E, Svensson M, Dorjsuren B, Togtmol M, Kauhanen J (2021). Does social health insurance prevent financial hardship in Mongolia? inpatient care: a case in point. PLoS One.

[31] Dugee O, Sugar B, Dorjsuren B, Mahal A (2019). Economic impacts of chronic conditions in a country with high levels of population health coverage: lessons from Mongolia. Trop Med Int Health.

[32] McClurkin MA, Yingling LR, Ayers C, Cooper-McCann R, Suresh V, Nothwehr A, Barrington DS, Powell-Wiley TM (2015). Health insurance status as a barrier to ideal cardiovascular health for U.S. adults: data from the National Health and Nutrition Examination Survey (NHANES). PLoS One.

[33] Crotti R, Pal KK, Ratcheva RZV (2021). Global gender gap report 2021. World Economic Forum.

[34] Appelman Y, van Rijn BB, Haaf MET, Boersma E, Peters SAE (2015). Sex differences in cardiovascular risk factors and disease prevention. Atherosclerosis.

[35] Colafella KMM, Denton KM (2018). Sex-specific differences in hypertension and associated cardiovascular disease. Nat Rev Nephrol.

[36] Regitz-Zagrosek V, Oertelt-Prigione S, Prescott E, Franconi F, Gerdts E, Foryst-Ludwig A, Maas AHEM, Kautzky-Willer A, Knappe-Wegner D, Kintscher U, Ladwig KH, Schenck-Gustafsson K, Stangl V, EUGenMed Cardiovascular Clinical Study Group (2016). Gender in cardiovascular diseases: impact on clinical manifestations, management, and outcomes. Eur Heart J.

[37] Tam HL, Leung LYL, Wong EML, Cheung K, Chan ASW (2022). Integration of text messaging interventions into hypertension management among older adults: a systematic review and meta-analysis. Worldviews Evid Based Nurs.

