# Supplementary material for: Sex and Age Differences in the Association Between Social Determinants of Health and Cardiovascular Health According to Household Income Among Mongolian Adults: Cross-Sectional Study
Source: JMIR Public Health Surveill. 2023 Dec 1;9:e44569. doi: 10.2196/44569 (PMC10724809; doi:10.2196/44569)
Supplement: Multimedia Appendix 1 [file publichealth_v9i1e44569_app1.docx]

| **Table S1**. Definition of cardiovascular health by modified Life’s Simple 7. | | | |
| --- | --- | --- | --- |
| CVH metrics component | Poor (0 point) | Intermediate (1 point) | Ideal (2 points) |
| Smoking | Current smoker | Former smoker (≤12 months) | Never smoked or quit smoking >12 months ago |
| Physical activity | None | Moderate intensity for  1–149 min/week or Vigorous intensity for 1–74 min/week or a combination of moderate and vigorous intensity for 1–149 min/week | Moderate intensity ≥150 min/week or Vigorous intensity ≥75 min/week or a combination both above ≥150 min/week |
| Salt consumption | Far too much or too much | Just the right amount | Far too little or too little |
| BMI^a^ | ≥25 kg/m^2^ | 23–<25 kg/m^2^ | <23 kg/m^2^ |
| SBP/DBP | SBP ≥ 140 mmHg or DBP ≥ 90 mmHg | SBP 120 –< 140 mmHg or DBP 80 –< 90 mmHg or treated to goal | SBP < 120 mmHg and DBP < 80 mmHg and not treated for HTN |
| Fasting plasma glucose | ≥126 mg/dL | 100–<126 mg/dL or treated to goal | <100 mg/dL and not treated for diabetes |
| Total serum cholesterol | ≥240 mg/dL | 200–<240 mg/dL or treated to goal | <200 mg/dL and not treated |
| Abbreviations: CVH, cardiovascular health; BMI, body mass index; SBP, systolic blood pressure; DBP, diastolic blood pressure. | | | |
| ^a^BMI was categorized using validated values for Asian populations. | | | |

| **Table S2.** Results of multinomial logistic regression about association between social determinants of health and CVH category by Life's simple 7 according to house income. | | | | | | | | | | | | | |
| --- | --- | --- | --- | --- | --- | --- | --- | --- | --- | --- | --- | --- | --- |
| Quartile of equivalized monthly house income | Social determinants of health | No. of intermediate CVH (%) | | OR (95% Confidence interval) | | | | No. of poor CVH (%) | | OR (95% Confidence interval) | | | |
| **Total** | **Education** |  |  |  |  |  |  |  |  |  |  |  |  |
| Q1, <77.5$ (N=1,274) | ≥12 years | 166 | (62.9) | 1.00 |  |  |  | 50 | (18.9) | 1.00 |  |  |  |
|  | <12 years | 625 | (61.9) | 1.53 | (0.89 | – | 2.64) | 254 | (25.2) | 2.42 | (1.30 | – | 4.51) |
|  |  |  |  |  |  |  |  |  |  |  |  |  |  |
| Q2, 77.5-137.2$ (N=1,263) | ≥12 years | 284 | (59.9) | 1.00 |  |  |  | 94 | (19.8) | 1.00 |  |  |  |
|  | <12 years | 447 | (56.7) | 1.49 | (0.92 | – | 2.42) | 270 | (34.2) | 1.90 | (1.08 | – | 3.33) |
|  |  |  |  |  |  |  |  |  |  |  |  |  |  |
| Q3, 137.2-232.8$ (N=1,280) | ≥12 years | 414 | (55.4) | 1.00 |  |  |  | 215 | (28.7) | 1.00 |  |  |  |
|  | <12 years | 273 | (51.3) | 0.55 | (0.34 | – | 0.89) | 200 | (37.6) | 0.58 | (0.35 | – | 0.96) |
|  |  |  |  |  |  |  |  |  |  |  |  |  |  |
| Q4, ≥232.8$ (N=1,279) | ≥12 years | 484 | (52.6) | 1.00 |  |  |  | 308 | (33.4) | 1.00 |  |  |  |
|  | <12 years | 197 | (55.0) | 0.88 | (0.52 | – | 1.50) | 120 | (33.5) | 0.70 | (0.40 | – | 1.22) |
| **Total** | **Health insurance** |  |  |  |  |  |  |  |  |  |  |  |  |
| Q1, <77.5$ (N=1,274) | Yes | 641 | (62.7) | 1.00 |  |  |  | 233 | (22.8) | 1.00 |  |  |  |
|  | No | 150 | (59.5) | 1.29 | (0.76 | – | 2.20) | 71 | (28.2) | 2.17 | (1.13 | – | 4.18) |
|  |  |  |  |  |  |  |  |  |  |  |  |  |  |
| Q2, 77.5-137.2$ (N=1,263) | Yes | 623 | (57.5) | 1.00 |  |  |  | 309 | (28.5) | 1.00 |  |  |  |
|  | No | 108 | (60.3) | 1.40 | (0.72 | – | 2.70) | 55 | (30.7) | 1.63 | (0.74 | – | 3.62) |
|  |  |  |  |  |  |  |  |  |  |  |  |  |  |
| Q3, 137.2-232.8$ (N=1,280) | Yes | 604 | (53.9) | 1.00 |  |  |  | 364 | (32.5) | 1.00 |  |  |  |
|  | No | 83 | (52.2) | 0.83 | (0.42 | – | 1.62) | 51 | (32.1) | 1.11 | (0.51 | – | 2.42) |
|  |  |  |  |  |  |  |  |  |  |  |  |  |  |
| Q4, ≥232.8$ (N=1,279) | Yes | 605 | (53.8) | 1.00 |  |  |  | 373 | (33.2) | 1.00 |  |  |  |
|  | No | 76 | (49.4) | 0.89 | (0.45 | – | 1.74) | 55 | (35.7) | 1.03 | (0.46 | – | 2.30) |
| Abbreviation: CVH, cardiovascular health; OR, odds ratio | | | | | | | | | | | | | |
| The estimates are in reference to the ideal CVH group. | | | | | | | | | | | | | |
| Age, sex, work status, area, history of heart attack or stroke, use of aspirin, and use of statin were adjusted. | | | | | | | | | | | | | |

| **Table S3.** Results of multinomial logistic regression about association between education level and CVH category according to house income by sex and age. | | | | | | | | |
| --- | --- | --- | --- | --- | --- | --- | --- | --- |
| Quartile of equivalized monthly house income | **Intermediate CVH** | | | | **Poor CVH** | | | |
|  | OR (95% Confidence interval) | | | | OR (95% Confidence interval) | | | |
| **Men** |  |  |  |  |  |  |  |  |
| Q1 (N=559) | 0.78 | (0.31 | – | 1.96) | 1.44 | (0.56 | – | 3.72) |
| Q2 (N=512) | 1.90 | (0.78 | – | 4.63) | 2.53 | (0.94 | – | 6.79) |
| Q3 (N=527) | 0.39 | (0.16 | – | 0.93) | 0.46 | (0.20 | – | 1.08) |
| Q4 (N=609) | 1.23 | (0.47 | – | 3.17) | 1.21 | (0.46 | – | 3.14) |
|  |  |  |  |  |  |  |  |  |
| **Women** |  |  |  |  |  |  |  |  |
| Q1 (N=715) | 2.31 | (1.25 | – | 4.27) | 2.99 | (1.35 | – | 6.63) |
| Q2 (N=751) | 1.35 | (0.76 | – | 2.39) | 1.64 | (0.77 | – | 3.52) |
| Q3 (N=753) | 0.64 | (0.35 | – | 1.17) | 0.58 | (0.30 | – | 1.13) |
| Q4 (N=670) | 0.78 | (0.41 | – | 1.47) | 0.33 | (0.14 | – | 0.80) |
|  |  |  |  |  |  |  |  |  |
| **18-44 years** |  |  |  |  |  |  |  |  |
| Q1 (N=715) | 1.70 | (0.95 | – | 3.05) | 3.22 | (1.54 | – | 6.72) |
| Q2 (N=640) | 1.54 | (0.89 | – | 2.68) | 2.23 | (1.17 | – | 4.27) |
| Q3 (N=741) | 0.57 | (0.33 | – | 0.99) | 0.96 | (0.55 | – | 1.69) |
| Q4 (N=792) | 0.97 | (0.54 | – | 1.77) | 1.03 | (0.56 | – | 1.91) |
|  |  |  |  |  |  |  |  |  |
| **45-69 years** |  |  |  |  |  |  |  |  |
| Q1 (N=559) | 0.84 | (0.28 | – | 2.52) | 1.14 | (0.34 | – | 3.85) |
| Q2 (N=623) | 1.63 | (0.73 | – | 3.63) | 2.03 | (0.84 | – | 4.90) |
| Q3 (N=539) | 0.41 | (0.17 | – | 0.99) | 0.25 | (0.10 | – | 0.60) |
| Q4 (N=487) | 0.59 | (0.22 | – | 1.57) | 0.36 | (0.13 | – | 0.97) |
| Abbreviation: CVH, cardiovascular health; OR, odds ratio | | | | | | | | |
| The estimates are in reference to the ideal CVH group. | | | | | | | | |
| ORs for <12 years of education were presented (Ref: ≥12 years of education). | | | | | | | | |
| Age, sex, work status, area, history of heart attack or stroke, use of aspirin, and use of statin were adjusted. | | | | | | | | |

| **Table S4.** Results of multinomial logistic regression about association between health insurance and CVH category according to house income by sex and age. | | | | | | | | |
| --- | --- | --- | --- | --- | --- | --- | --- | --- |
| Quartile of equivalized monthly house income | **Intermediate CVH** | | | | **Poor CVH** | | | |
|  | OR (95% Confidence interval) | | | | OR (95% Confidence interval) | | | |
| **Men** |  |  |  |  |  |  |  |  |
| Q1 (N=559) | 1.54 | (0.58 | – | 4.07) | 2.02 | (0.73 | – | 5.57) |
| Q2 (N=512) | 1.61 | (0.50 | – | 5.18) | 1.54 | (0.47 | – | 5.08) |
| Q3 (N=527) | 0.85 | (0.29 | – | 2.47) | 1.43 | (0.52 | – | 3.96) |
| Q4 (N=609) | 0.40 | (0.16 | – | 0.99) | 0.42 | (0.16 | – | 1.07) |
|  |  |  |  |  |  |  |  |  |
| **Women** |  |  |  |  |  |  |  |  |
| Q1 (N=715) | 1.13 | (0.57 | – | 2.24) | 2.54 | (1.09 | – | 5.90) |
| Q2 (N=751) | 1.22 | (0.55 | – | 2.69) | 2.30 | (0.74 | – | 7.10) |
| Q3 (N=753) | 1.10 | (0.45 | – | 2.68) | 1.00 | (0.34 | – | 2.94) |
| Q4 (N=670) | 1.52 | (0.60 | – | 3.84) | 2.39 | (0.88 | – | 6.48) |
|  |  |  |  |  |  |  |  |  |
| **18-44 years** |  |  |  |  |  |  |  |  |
| Q1 (N=715) | 1.14 | (0.63 | – | 2.07) | 2.03 | (0.98 | – | 4.18) |
| Q2 (N=640) | 1.23 | (0.60 | – | 2.51) | 1.49 | (0.59 | – | 3.72) |
| Q3 (N=741) | 0.75 | (0.37 | – | 1.54) | 1.11 | (0.47 | – | 2.60) |
| Q4 (N=792) | 0.84 | (0.42 | – | 1.69) | 0.90 | (0.39 | – | 2.08) |
|  |  |  |  |  |  |  |  |  |
| **45-69 years** |  |  |  |  |  |  |  |  |
| Q1 (N=559) | 1.87 | (0.45 | – | 7.81) | 2.67 | (0.60 | – | 11.95) |
| Q2 (N=623) | 6.39 | (0.83 | – | 49.41) | 6.05 | (0.73 | – | 50.23) |
| Q3 (N=539) | NA |  |  |  | NA |  |  |  |
| Q4 (N=487) | 3.57 | (0.38 | – | 33.85) | 6.61 | (0.63 | – | 69.40) |
| Abbreviation: CVH, cardiovascular health; OR, odds ratio | | | | | | | | |
| The estimates are in reference to the ideal CVH group. | | | | | | | | |
| ORs for absence of health insurance were presented (Ref: have health insurance). | | | | | | | | |
| Age, sex, work status, area, history of heart attack or stroke, use of aspirin, and use of statin were adjusted. | | | | | | | | |

| **Table S5.** Results of multinomial logistic regression about association between social determinants of health and CVH category according to house income by area. | | | | | | | | | | |
| --- | --- | --- | --- | --- | --- | --- | --- | --- | --- | --- |
| Social determinants of health | Area | Quartile of equivalized monthly house income | **Intermediate CVH** | | | | **Poor CVH** | | | |
|  |  |  | OR (95% Confidence interval) | | | | OR (95% Confidence interval) | | | |
| Education level (Ref: ≥12 years of education) | Rural | Q1 (N=681) | 1.74 | (0.76 | – | 3.96) | 3.22 | (1.16 | – | 8.91) |
|  |  | Q2 (N=442) | 2.86 | (1.49 | – | 5.49) | 3.94 | (1.65 | – | 9.45) |
|  |  | Q3 (N=319) | 1.07 | (0.37 | – | 3.12) | 0.77 | (0.23 | – | 2.57) |
|  |  | Q4 (N=233) | 1.34 | (0.41 | – | 4.41) | 1.10 | (0.34 | – | 3.55) |
|  | Urban | Q1 (N=593) | 1.37 | (0.65 | – | 2.89) | 1.94 | (0.85 | – | 4.42) |
|  |  | Q2 (N=821) | 1.15 | (0.62 | – | 2.13) | 1.43 | (0.71 | – | 2.90) |
|  |  | Q3 (N=961) | 0.47 | (0.28 | – | 0.80) | 0.53 | (0.30 | – | 0.92) |
|  |  | Q4 (N=1,046) | 0.80 | (0.44 | – | 1.43) | 0.68 | (0.36 | – | 1.27) |
| Health insurance (Ref: have health insurance) | Rural | Q1 (N=681) | 1.30 | (0.66 | – | 2.53) | 2.88 | (1.25 | – | 6.68) |
|  |  | Q2 (N=442) | 1.42 | (0.44 | – | 4.59) | 1.48 | (0.40 | – | 5.42) |
|  |  | Q3 (N=319) | 2.24 | (0.40 | – | 12.64) | 5.43 | (0.74 | – | 39.85) |
|  |  | Q4 (N=233) | 0.78 | (0.15 | – | 4.02) | 1.76 | (0.26 | – | 11.91) |
|  | Urban | Q1 (N=593) | 1.21 | (0.50 | – | 2.97) | 1.25 | (0.45 | – | 3.48) |
|  |  | Q2 (N=821) | 1.33 | (0.59 | – | 3.04) | 1.64 | (0.59 | – | 4.52) |
|  |  | Q3 (N=961) | 0.66 | (0.31 | – | 1.37) | 0.69 | (0.30 | – | 1.60) |
|  |  | Q4 (N=1,046) | 0.93 | (0.45 | – | 1.95) | 0.84 | (0.36 | – | 1.96) |
| Abbreviation: CVH, cardiovascular health; OR, odds ratio | | | | | | | | | | |
| The estimates are in reference to the ideal CVH group. | | | | | | | | | | |
| Age, sex, work status, history of heart attack or stroke, use of aspirin, and use of statin were adjusted. | | | | | | | | | | |
