# Supplementary material for: Sex and Age Differences in the Association Between Social Determinants of Health and Cardiovascular Health According to Household Income Among Mongolian Adults: Cross-Sectional Study
Source: JMIR Public Health Surveill. 2023 Dec 1;9:e44569. doi: 10.2196/44569 (PMC10724809; doi:10.2196/44569)
Supplement: Multimedia Appendix 2 [file publichealth_v9i1e44569_app2.docx]

**Table S1.** General characteristics of study participants according to CVH by modified Life's Simple 7.

| Variables | Total (N=5,691) | | Men | | | | | | P-value | Women | | | | | | P-value | |
| --- | --- | --- | --- | --- | --- | --- | --- | --- | --- | --- | --- | --- | --- | --- | --- | --- | --- |
|  |  |  | Poor CVH (N=1,036) | | Intermediate CVH (N=1,265) | | Ideal CVH (N=220) | |  | Poor CVH (N=649) | | Intermediate CVH (N=1,943) | | Ideal CVH (N=578) | |  |  |
| Age, years | 37.5 | (36.9-38.0) | 42.4 | (41.5-43.4) | 36.0 | (35.0-36.9) | 27.0 | (25.7-28.2) | <.001 | 44.3 | (42.9-45.7) | 38.9 | (38.1-39.7) | 30.1 | (29.1-31.1) | <.001 |  |
| 18-39 | 2,608 | (59.0) | 353 | (42.8) | 674 | (65.2) | 166 | (87.6) | <.001 | 168 | (37.0) | 841 | (55.1) | 464 | (88.4) | <.001 |  |
| 40-59 | 2,387 | (34.4) | 532 | (47.6) | 469 | (29.3) | 47 | (11.0) |  | 349 | (51.2) | 842 | (37.8) | 114 | (11.6) |  |  |
| ≥ 60 | 696 | (6.5) | 151 | (9.6) | 122 | (5.4) | 7 | (1.5) |  | 132 | (11.7) | 260 | (7.1) | 24 | (1.6) |  |  |
| < 12 years of education | 3,075 | (49.6) | 631 | (58.3) | 768 | (54.2) | 127 | (49.7) | 0.100 | 333 | (46.9) | 982 | (46.4) | 234 | (36.8) | 0.001 |  |
| Health insurance (no) | 888 | (19.5) | 189 | (20.9) | 251 | (21.5) | 55 | (29.3) | 0.078 | 81 | (17.4) | 239 | (15.6) | 73 | (19.4) | 0.246 |  |
| Quartile of household income (monthly)^a^ |  |  |  |  |  |  |  |  | 0.001 |  |  |  |  |  |  | 0.287 |  |
| Q1 (lowest) | 1,274 | (25.2) | 176 | (18.7) | 328 | (29.5) | 55 | (28.7) |  | 128 | (20.8) | 463 | (26.4) | 124 | (24.8) |  |  |
| Q2 | 1,263 | (23.5) | 213 | (22.4) | 263 | (22.1) | 36 | (19.7) |  | 151 | (24.7) | 468 | (25.1) | 132 | (24.9) |  |  |
| Q3 | 1,280 | (24.8) | 235 | (25.4) | 250 | (22.2) | 42 | (28.5) |  | 180 | (30.3) | 437 | (24.1) | 136 | (25.8) |  |  |
| Q4 (highest) | 1,279 | (26.4) | 293 | (33.5) | 275 | (26.2) | 41 | (23.2) |  | 135 | (24.2) | 406 | (24.4) | 129 | (24.5) |  |  |
| Rural | 2,015 | (36.6) | 368 | (35.3) | 526 | (40.7) | 94 | (42.4) | 0.121 | 197 | (33.8) | 629 | (33.4) | 201 | (36.8) | 0.511 |  |
| Work status |  |  |  |  |  |  |  |  | <.001 |  |  |  |  |  |  | 0.010 |  |
| Employee | 2,149 | (37.5) | 375 | (36.9) | 461 | (36.4) | 57 | (23.4) |  | 224 | (38.4) | 774 | (41.7) | 258 | (37.2) |  |  |
| Self-employed | 1,495 | (26.0) | 348 | (35.0) | 443 | (33.8) | 73 | (24.9) |  | 108 | (17.8) | 408 | (20.5) | 115 | (16.3) |  |  |
| Others^b^ | 2,047 | (36.5) | 313 | (28.1) | 361 | (29.8) | 90 | (51.8) |  | 317 | (43.8) | 761 | (37.8) | 205 | (46.5) |  |  |
| Use of aspirin | 852 | (11.7) | 182 | (14.6) | 139 | (9.4) | 14 | (4.7) | <.001 | 153 | (18.9) | 316 | (13.1) | 48 | (6.8) | <.001 |  |
| Use of statin | 177 | (2.5) | 31 | (2.2) | 22 | (1.3) | 2 | (2.2) | 0.464 | 37 | (4.3) | 80 | (3.9) | 5 | (0.5) | <.001 |  |
| History of heart attack or stroke | 936 | (15.1) | 179 | (16.0) | 167 | (12.2) | 31 | (12.4) | 0.123 | 130 | (17.8) | 335 | (17.0) | 94 | (14.2) | 0.291 |  |
| Abbreviations: CVH, cardiovascular health | | | | | | | | | | | | | | | | | |
| Values are presented as weighted mean (95% CI) or number (weighted %). | | | | | | | | | | | | | | | | | |
| ^a^Sum of numbers can miss the total number in group due to missing values. Q1: <US $77.5 (=230470 MNT), Q2: US $77.5-137.2 (=408248 MNT), Q3: US $137.2-232.8 (=692820 MNT), Q4: ≥US $232.8 | | | | | | | | | | | | | | | | | |
| ^b^Includes non-paid, student, homemaker, retired, or unemployed. | | | | | | | | | | | | | | | | | |
